# Functional MRI Changes in Patients after Thyroidectomy under General Anesthesia

**DOI:** 10.1155/2022/1935125

**Published:** 2022-06-21

**Authors:** Xilun Yang, Bing Yu, Ling Ma

**Affiliations:** ^1^Department of Anesthesiology, Shengjing Hospital of China Medical University, China; ^2^Department of Radiology, Shengjing Hospital of China Medical University, China

## Abstract

Cognitive changes affecting elderly patients following surgery under anesthesia have drawn significant attention and have been investigated in considerable depth. Resting-state functional magnetic resonance imaging (rs-fMRI) can be used to assess changes in brain functional connectivity (FC) associated with postoperative changes in cognition, a common complication in seniors undergoing surgery. In this study, we recruited 20 patients over 55 of age and scheduled an elective thyroidectomy under general anesthesia to assess perioperative changes in brain FC density (FCD) in patients undergoing thyroidectomy under general anesthesia using rs-fMRI. All 20 patients underwent a series of clinical, quantitative, neurological, and neuropsychological tests and fMRI examinations on the day before surgery (Day 0) and 7 days after surgery (Day 7). The following tests were conducted on all patients: the Minimental State Examination (MMSE), the digit symbol substitution test (DSST), the trail making test (part A), the verbal fluency test, and Warrington's recognition memory test (WRMT). FMRI data were acquired using a 3T MR system; the FCD values were calculated using the REST software package. We used paired *t*-tests to compare the FCD between Day 7 and Day 0. A value of *p* < 0.05 was considered to reflect statistical significance. The postoperative FCD was significantly reduced in the supplementary motor area (SMA). Analyses of the percentage changes of errors in the WRMT revealed a significant and negative correlation with the mean percentage change of FCD in the SMA (Spearman's *r* = −0.54, 95% CI: (-0.80, -0.12), *p* = 0.014). Postoperative changes in FCD in the SMA may be associated with the perioperative neurocognitive changes in patients undergoing partial thyroidectomy under general anesthesia.

## 1. Introduction

General anesthesia plays an important role in ensuring that operations proceed smoothly. However, a number of complications may occur after general anesthesia, including perioperative neurocognitive disorders (PNDs) [1]. PND predominantly occurs in elderly patients and is associated with an increased risk of mortality and a longer period of welfare benefits.

The pathophysiological mechanisms of PND have been assessed in great depth in recent years. These studies were mainly carried out using animal models, suggesting that research should focus on the comprehensive effects of the combined use of anesthetics and surgical procedures.

The neurotoxicity of anesthetics has drawn significant attention among both pediatric patients and adults and has been investigated in significant depth. For example, Hu et al. showed that children undergoing multiple exposures were more likely to develop adverse effects related to learning and attention [2]. However, three major retrospective cohort studies demonstrated that exposure to single or multiple anesthetics did not increase the possibility of postoperative cognitive disorders [3–5]. Another study investigated the long-term adverse cognitive effects of surgery combined with anesthesia using data from a cohort of 8,503 middle-aged and elderly twins [6], showing that the underlying disease played a more important role in cognitive functionality in mid- and later life than surgery and anesthesia per se [6]. A meta-analysis of adult patients further showed that general anesthesia may increase the risk of developing postoperative cognitive dysfunction (POCD), but not postoperative delirium (POD) [7]. There remains a certain degree of controversy regarding the effect of anesthetics on brain function and cognition [8].

Some functional magnetic resonance imaging (fMRI) studies have also focused on pre- and postoperative changes in brain function. Resting-state fMRI (rs-fMRI), based on the measurement of low-frequency fluctuations in blood oxygen level-dependent (BOLD) signals, is a noninvasive imaging technique used to measure brain activity. It has become a useful tool to investigate the brain's functional connectivity (FC) [9–15]. FC density (FCD), a fundamental fMRI parameter, has been widely used to describe local and network properties related to brain function at a resting state [16]. Furthermore, it is able to integrate information at the level of the entire brain, conducive to successfully quantifying abnormalities in the structural and functional network properties associated with schizophrenia, Alzheimer's disease, attention deficit hyperactivity disorder, epilepsy, and other disorders [17]. The brain of middle- and old-aged patients is more susceptible to changes in response to anesthesia, anesthetics, surgical insults, inflammation, hemodynamic changes, and postoperative pain. Indeed, these events can result in changes of mental state, social activities, and cognitive ability [18, 19]. Neuroimaging studies of PND that focus predominantly on anesthetics have yet to be carried out.

This prospective and observational study is aimed at investigating changes in the brain FCD before and 7 days following surgery using fMRI. In the current study, we selected patients with nonfunctional goiter undergoing partial thyroidectomy under general anesthesia to minimize any interference resulting from patient hemorrhage, major surgical procedures, and prolonged periods of hypothermia.

## 2. Materials and Methods

### 2.1. Subjects

The Medical Ethics Committee of Shengjing Hospital of China Medical University approved the study protocol (reference number: 2014PS149K). The study was also registered with the Chinese Ethics Committee of Registered Clinical Trials (registration number: ChiCTR-DDD-17014002). Informed written consent was obtained from all participants. We prospectively enrolled patients aged 55 or older who were diagnosed with nonfunctional goiter and underwent partial thyroidectomy (lobectomy/isthmusectomy) under general anesthesia between June 2014 and June 2015.

The inclusion criteria were as follows: (1) patients over 55 years of age, (2) patients with a physical status classification of American Society of Anesthesiologists (ASA) I-II, (3) patients who were right-handed, (4) anesthesia time lasting between 1 and 4 hours, and (5) blood loss < 400 mL. The exclusion criteria were as follows: (1) preoperative diseases of the nervous system, (2) a history of receiving psychotropic drugs or neurosurgery, (3) preoperative pathological changes of the major organs and systems, (4) uncontrolled preoperative diabetes, (5) preoperative hypertension with a blood pressure ≥ 140/90 mmHg, (6) head movement > 2 mm during MRI scanning, (7) the preoperative Minimental State Examination (MMSE) [20] <24, and (8) perioperative persistent hypertension or arrhythmia.

### 2.2. Anesthetic Management

Patients received no preoperative medication. Sufentanil (0.3 *μ*g·kg^−1^), etomidate (0.2 mg·kg^−1^), and cisatracurium (0.2-0.3 mg·kg^−1^) were administered intravenously to induce rapid anesthesia. This was followed by mechanical ventilation after endotracheal intubation. The anesthesia machine parameter settings were as follows: inhaled O_2_ : air = 1 : 1; a flow rate of 2.0 L/min; a tidal volume of 8-10 mL/kg; an inhalation time : exhalation time of 1 : 2; a respiratory rate of 10-16 times/min; a pulse oxygen saturation of 98%-100%; and an EtCO_2_ of 35-45 mmHg. Propofol (3–4 mg·kg^−1^·h^−1^), remifentanil (0.05–2 *μ*g·kg^−1^·h^−1^), and 2% sevoflurane were used to maintain MAC at 0.8-1.0 and BIS at 40-60. Blood pressure and heart rate were maintained at ±20% around baseline, and perioperative body temperature was kept between 36°C and 37°C.

### 2.3. fMRI Scanning, Data Acquisition, and Statistical Analysis

#### 2.3.1. Magnetic Resonance Data Acquisition

All patients were subjected to fMRI scanning at 7 am on the day of surgery and on postoperative day 7. fMRI scanning was carried out using a Philips Intera Ingenia 3.0T superconducting magnetic resonance scanning system with a 15-channel phase-sensitive encoding (SENSE) head coil. Resting-state BOLD data were acquired with a single shot GRE-EPI sequence; TR/TE was 2000/30 ms; FOV was 230 mm × 230 mm; the matrix was 64 × 64 with 28 layers and a layer thickness of 4 mm; flip angle (FA) was 90°, and scan time lasted 488 s, with the scanning plane parallel to the anterior connection and posterior connection (AC-PC), while scanning from the top of the skull to the medulla. Prior to each examination, an 8-second dummy scan was carried out to stabilize the magnetic field. During the scanning process, the patients were asked to close their eyes and keep quiet, to avoid thinking, and to avoid movement of the body above the shoulders. The patient's head was fixed by a sponge pad on both sides of the head as best as possible, and the ears were protected with earplugs to reduce any external noise.

#### 2.3.2. Data Processing

First, the original data was converted into NIFTI format, after which the functional data was subjected to preprocessed by SPM12 software provided within the MATLAB R2016 platform (MathWorks, Natick, MA, USA). The fMRI data were first realigned to correct head motion. Participants were excluded if their maximum head translation exceeded 2 mm on each axis or if their maximum angular motion exceeded 2.0° for each axis. For each fMRI session, the framewise displacement (FD) was also calculated. Corrections of acquisition time delay were then conducted. The resulting images were then spatially normalized to Montreal Neurological Institute- (MNI-) labeled space and resampled to 3.5 × 3.5 × 4 mm voxels. The normalized images were spatially smoothened with an 8 mm isotropic full-width at a half-maximum 3-D Gaussian kernel. Smoothened rs-fMRI data were analyzed using the rs-fMRI data analysis toolkit (REST) for wave filtering and linear drift removal. Signals of 0.01-0.08 Hz were analyzed to minimize the influence of low-frequency linear drift and high-frequency respiration and heartbeat noise.

#### 2.3.3. FCD Analysis

Using the pretreated graph, we extracted a time series for all voxels using REST software and calculated Pearson's correlation coefficient between each voxel, as shown in

(1) $$\begin{array}{c} R=\left[rij\right],\;i,j=1,2,\cdots ,N. \end{array}$$

In Equation (1), *N* represents the number of all voxels and *rij* represents Pearson's coefficient of any voxels *i* and *j* in the time series.

In the weighted brain network,

(2) $$\begin{array}{c} aij=\left\{\begin{array}{cc} 0, & rij\leq rth,\\ zij, & rij>rth, \end{array}\right. \end{array}$$

where *rth* represents whether there is a threshold in functional connectivity. In this study, *rth* was set to 0.25. The voxel with *r*_*ij*_ ≤ *r*_*th*_ was deleted to avoid the interference of noise and head movement on FC. *z*_*ij*_ represents the Fisher's transformation of correlation to *z*-score. This way, the connection of the network *a*_*ij*_ has a normal distribution and it simplifies the subsequent data analysis.

(3) $$\begin{array}{c} ki=\frac{1}{N-1}\underset{j\neq i}{\sum }aij. \end{array}$$

FCD is the most commonly used graphical method to recognize the core center in the whole brain. In Equation (3), *N* represents the total number of voxels and *k*_*i*_, the FCD of each voxel, and resembles the mean FC between voxel *i* and all other voxels; this can represent the influential strength of each voxel *i* within the brain network. The FCD reflects the information integration ability of voxels at the level of the whole brain. A larger voxel FCD implies that the voxel has a larger effect on the integration of information in the whole brain. The FCD *k*_*i*_ of each voxel in each patient in this study was calculated and plotted for both the preoperative scanned images and the postoperative scanned images.

### 2.4. Neurocognitive Function Assessment

All eligible participants underwent a series of clinical, quantitative, neurological, and neuropsychological tests that were performed by a qualified psychologist on the day before surgery and seven days after surgery. According to the International Study of Postoperative Cognitive Dysfunction (ISPOCDI and ISPOCD2) [21, 22], the series of tests (focused on memory, learning, attention, executive functions, and cognitive flexibility) included the MMSE, the digit symbol substitutions test (DSST) [23], the trail making test (part A) [24], the verbal fluency test [25], and Warrington's recognition memory test (WRMT) [26, 27]. The percentage change of score for each individual test was calculated using the equation (test score 1 week after surgery the preoperative baseline score)/baseline score.

### 2.5. Perioperative Thyroid Function

Thyroidectomy status, thyroid function, including free triiodothyronine (FT3), free thyroxine (FT4), and thyroid-stimulating hormone (TSH) were routinely assessed preoperatively and on the 7^th^ day after surgery.

### 2.6. Data Analysis

SPSS 17.0 software (SPSS Inc., Chicago, IL, USA) was used to analyze the general demographic data, and results were expressed as mean ± standard deviation. Preoperative and postoperative FCD were compared using paired *t*-tests using the SPM12 (https://www.fil.ion.ucl.ac.uk/spm) software package. The comparative results were corrected for multiple comparisons to a significance level of *p* < 0.05 by the REST AlphaSim program with 10,000 iterations combining an individual voxel threshold *p* < 0.001 with a minimum cluster size > 10 voxels. The preoperative and postoperative FDs were also compared using paired *t*-tests.

If any significant differences between preoperative and postoperative FCD were identified in any clusters, the mean percentage changes of FCD in those clusters were then calculated. Spearman's analysis was also performed to probe the relationship between the percentage changes of score for each individual neuropsychological test and the mean FCD. A value of *p* < 0.05 was considered statistically significant.

## 3. Results

**Figure 1:**
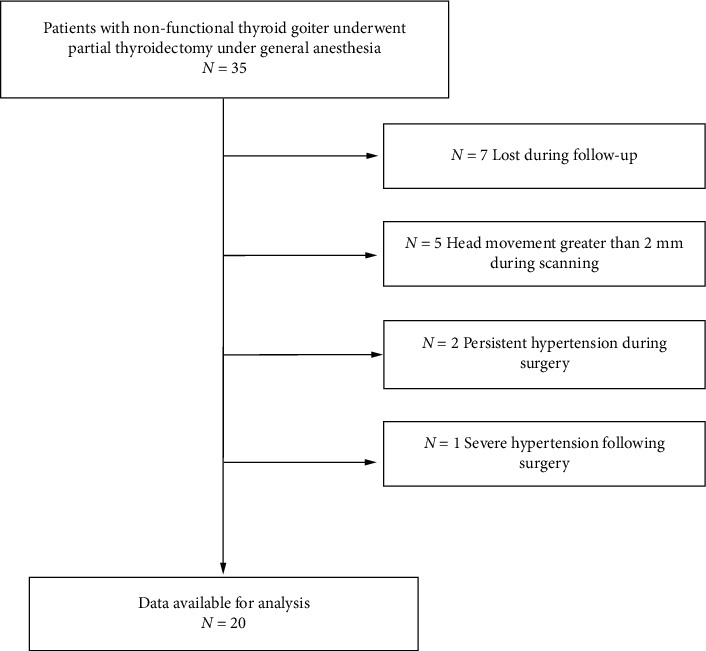
Flow chart representing patient enrolment.

We recruited a total of 35 patients, with seven cases lost during follow-up, five cases excluded due to a head movement > 2 mm during scanning, and two cases excluded due to persistent hypertension during surgery due to lack of control. One other case was excluded as a result of severe hypertension following surgery. Finally, we used data from 20 cases in our final statistical analysis (Figure 1).

### 3.1. Demographic Data and Cognitive Outcomes

The mean age of the enrolled patients was 63.1 ± 5.03 years with 12 male and 8 female patients. The mean educated years were 7.50 ± 3.47 years. The mean anesthesia time lasted 142.30 ± 75.49 minutes, and the mean blood loss during surgery was 54.00 ± 77.83 mL (Table 1). Comparison between preoperative mean arterial pressure (94.0 ± 4.4 mmHg), body temperature (36.4 ± 0.2°C), hemoglobin (12.9 ± 1.0 g/dL) with mean arterial pressure on the 7^th^ postoperative day (93.4 ± 4.5 mmHg), body temperature on the 7^th^ postoperative day (36.4 ± 0.2°C), and hemoglobin on the 7^th^ postoperative day (12.9 ± 1.0 g/dL) showed no significant differences, *p* > 0.05). Postoperative, the MMSE score decreased by 1.3 ± 0.1%, DSST decreased by 6.8 ± 0.9%, trail making test (part A) score increased by 1.1 ± 0.4%, verbal fluency test decreased by 2.4 ± 0.3%, and WRMT error score increased by 8.3% (0-33.3) (Table 2).

### 3.2. Radiological Results

**Figure 2:**
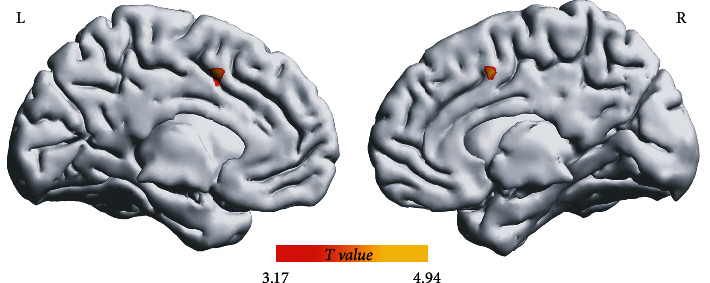
Postoperative FCD changes the postoperative FCD decreased significantly in the supplementary motor area (SMA) (peak MNI coordinates: *x* = 0, *y* = 9, *z* = 45; cluster size = 23 voxels; peak *T* = 4.9438, *p* < 0.001, AlphaSim corrected).

**Figure 3:**
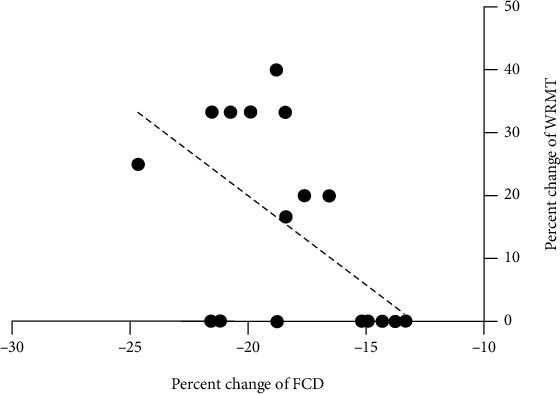
Correlation of changes in WRMT and FCD. The percentage changes in errors in the WRMT revealed a significant and negative correlation with the mean percentage changes of FCD in the SMA (Spearman's *r* = 0.54, 95% CI: (-0.80, -0.12), *p* = 0.014).

The postoperative FCD decreased significantly in the supplementary motor area (SMA). (Peak MNI coordinates: *x* = 0, *y* = 9, *z* = 45; cluster size = 23 voxels; peak *T* = 4.94, *p* < 0.001, AlphaSim corrected, Figure 2). Analyses of the percentage changes of errors in the WRMT revealed a significant and negative correlation with the mean percentage changes of FCD in the SMA (Spearman's *r* = −0.54, 95% CI: (-0.80, -0.12), *p* = 0.014, Figure 3).

### 3.3. Thyroid Function Changes

Their preoperative FT3, FT4, and TSH were all within a normal range. Their FT3, FT4, and TSH 7 days after operation were all within a normal range. No significant changes were observed after patients' thyroidectomy operations (Table 3).

## 4. Discussion

Except for surgical trauma and immune responses, the application of anesthetics and the mode of anesthesia may influence perioperative neural functionality. In our study, we chose to focus on a partial thyroidectomy, as this type of surgery is usually associated with lower blood loss, less complicated surgical procedures, and fewer postoperative complications. All patients in this study were diagnosed with a nonfunctional goiter, which means that fewer extraneous factors could have influenced the effect of surgery and anesthesia on brain FCD. In our current study, we found that FCD decreased significantly in the SMA and that the changes of FCD in the SMA were correlated with score changes in the WRMT.

Our study also revealed that the WRMT scores changed more significantly than other neurocognitive function test scores. This result was similar to that which was described by Whitlock et al. [28] and Browndyke et al. [29]. Given that none of the patients experienced massive perioperative hemorrhage, long-term cerebral hypoxia, or cerebral ischemia, we speculate that the perioperative cognitive changes mainly be related to central neuroinflammatory responses [30]. Further studies on inflammatory mediators are warranted to confirm this speculation.

FC is aimed at assessing the connection between two spatial regions of interest under the assistance of linear temporal correlation. There are many methods to analyze the brain FC, such as a functional connection analysis based on seed points (regions of interest, ROI) [31], a functional connection analysis based on voxels [32], regional homogeneity (ReHo) tests [33], and amplitude low-frequency fluctuation (ALFF) assessments [34]. The ROI analysis is restricted by the choice of seed points. In other words, different choices of seed points lead to different results. ReHo and ALFF measure the spontaneous activity of local neurons and reflect local, short-distance brain FC. This study analyzed the FC based on the voxel of the entire brain.

Brain functional changes during and after surgery under general anesthesia have long been investigated. Based on fMRI FC analyses between the preoperative baseline and 6 weeks after operation, Browndyke et al. reported postoperative changes in global cognitive function in the DMN-associated posterior cingulate cortex (PCC)/precuneus and the right superior frontal gyrus (rSFG) cortical regions following major cardiac surgery [35]. In another study, Browndyke et al. studied patients undergoing major cardiac surgery using a block-designed verbal N-back working memory task prior to and during MRI scanning [29]. They demonstrated that the 6-week postoperative working memory load-related FCD increased in the left dorsal PCC (dPCC). The increases in dPCC, FCD, and local coherence were inversely associated with global cognitive changes in surgical patients but not in healthy controls. Haiqing et al. compared fMRI pre- and postoperatively within 48 h of surgery, revealing significant reductions in rs-fMRI connectivity for the default mode network (DMN), silence network (SN), and central executive network (CEN) in nondemented older adults undergoing unilateral total knee arthroplasty under general anesthesia with continuous femoral and single-injection subgluteal sciatic nerve blocks [36]. In another study, Lan et al. found that reduced amplitude of low-frequency fluctuations (ALFF) persisted in the left precuneus gyrus and middle temporal gyrus 1 week after total knee arthroplasty under spinal anesthesia when compared with preoperative data [37]. However, these studies assessed perioperative changes following major surgery; these operations are often associated with multiple factors that could result in changes in neural function, including massive hemorrhage and long periods of low temperature. In contrast, our current study in patients with normal perioperative thyroid function undergoing partial thyroidectomy showed the FCD decreased in SMA up to 7 days postsurgically.

One of the first neuroimaging investigations by White and Alkire [38] also reported an impairment of interactions that predominantly involved the primary motor and supplementary motor association cortices during isoflurane or halothane anesthesia. The term “SMA” was introduced in the 1950s [39]. The SMA occupies the posterior third of the superior frontal gyrus and constitutes the medial region of Brodmann's area 6 [40, 41]. The SMA is connected to the limbic system, basal ganglia, cerebellum, thalamus, contralateral SMA, superior parietal lobe, and portions of the frontal lobes via fiber tracts [42, 43]. These regions' main functions are to connect the scattering functional regions and coordinate the integral manifestation of the brain [44]. Functional studies have suggested that the SMA is involved in the planning and preparation of movements. The SMA also has higher-order functions in language production, recognition of movement and thinking, memory storage, the establishment of visual and motor relationships, learning, perception of time, intention of action, conflict resolution, and transition between actions [45]. The SMA is known to be crucial to multiple aspects of motor behavior, including action preparation, initiation and selection of actions, motor learning, inhibition, conditional actions, action control, and the monitoring of action outcomes [46]. The SMA has also been implicated in other cognitive and motor functions, such as learning new associations between stimuli and responses at the beginning of a new action or during the inhibition of response [47]. The SMA plays a role in facilitating spontaneous motor responses to sound and in supporting a flexible engagement of sensorimotor processes to enable imagery and to guide auditory perception [48]. Based on functional studies, the SMA has been found to participate in the activation, control, and generation of movement and is tightly coupled to cognitive, nonmotor tasks. An intraoperative electrostimulation study demonstrated that the somatotopic organization of the SMA is related to the inferior limb, superior limb, and the face, distributed from posterior to anterior [49]. A fMRI study carried out with 0.25 MAC sevoflurane in healthy ASA volunteers showed there was a statistically significant decrease in activation with sevoflurane in the thalamus, hippocampus, and SMA [50], which is partly consistent with our findings.

There are a number of limitations to this study that need to be taken into consideration. First, the postoperative changes of brain FCD in middle-aged and elderly patients can be caused by many factors, including surgical trauma, application of anesthetics, and inflammation. In addition, we failed to consider all independent factors that could influence postoperative brain function. Second, this is a prospective and observational study. Third, the changes in FCD and cognitive function were the results of both surgery and anesthesia, since nowadays, the surgery is almost taken under anesthesia. We cannot conclude the result is caused by surgery or anesthesia alone. Fourth, the number of patients included in the study was small and the follow-up period was relatively short. Therefore, future studies involving larger groups of patients in multiple centers are warranted.

In conclusion, the brain FC network in middle-aged and elderly patients following partial thyroidectomy under general anesthesia revealed a reduction in the FCD of the SMA, and this reduction is correlated with the percentage change of error in WRMT following an operation.

## Figures and Tables

**Table 1:** Enrolled patients' demographic characteristics.

Age (years)	63.1 ± 5.03
Gender (male/female)	12/8
Educated period (years)	7.50 ± 3.47
Anesthesia time (min)	142.30 ± 75.49
Blood loss (mL)	54.00 ± 77.83

Data are presented as mean ± standard deviation.

**Table 2:** Changes in neuropsychological test scores.

	Day 0	Day 7	Percent change (%)
MMSE	29.1 ± 0.7	28.7 ± 0.7	−1.3 ± 0.1
DSST	32.2 ± 4.6	30.0 ± 4.4	−6.8 ± 0.9
Trail making test (part A)	17.6 ± 5.5	17.8 ± 5.8	+1.1 ± 0.4
Verbal fluency test	16.8 ± 1.8	16.4 ± 1.9	−2.4 ± 0.3
WRMT	1.3 (0.8-1.9)	1.5 (0.8-2.0)	+8.3 (0-33.3)

Data are presented as median (IQR) or mean ± standard deviation.

**Table 3:** Free T3, T4, and TSH in the studied patients.

	Day 0	Day 7	*p*
FT3 (pmol/L)	4.08 ± 0.98	4.07 ± 0.97	0.418
FT4 (pmol/L)	10.50 ± 3.32	10.49 ± 3.33	0.656
TSH (mIU/mL)	2.35 ± 1.41	2.33 ± 1.42	0.406

Data are presented as mean ± standard deviation.

## Data Availability

The data used to support the findings of this study are available from the corresponding author upon request.
